# Apogossypolone Inhibits Cell Proliferation and Epithelial-Mesenchymal Transition in Cervical Cancer *via* Activating DKK3

**DOI:** 10.3389/fonc.2022.948023

**Published:** 2022-07-18

**Authors:** Yuling Li, Jinfeng Qu, Lu Liu, Yu Sun, Junhua Zhang, Sai Han, Youzhong Zhang

**Affiliations:** ^1^ Department of Obstetrics and Gynecology, Qilu Hospital of Shandong University, Jinan, China; ^2^ Department of Obstetrics and Gynecology, Central Hospital Affiliated to Shandong First Medical University, Jinan, China

**Keywords:** cervical cancer, apogossypolone, DKK3, apoptosis, EMT

## Abstract

Apogossypolone (ApoG2), a novel derivative of gossypol lacking of two aldehyde groups, exhibits anti-tumor effects. However, the mechanisms by which ApoG2 regulates cervical cancer (CC) cells remain unclear. In this study, we treated two CC cell lines (CaSki and HeLa) with an increasing concentration of ApoG2 for 24 h. Cell Counting Kit-8 (CCK-8) assay, colony formation assay, flow cytometry and transwell invasion assay were utilized to detect cell proliferation, apoptosis and invasion *in vitro*. We first observed that ApoG2 inhibited cell proliferation, invasion and epithelial-to-mesenchymal transition (EMT) process in CC cells, along with upregulation of Dickkopf Wnt signaling pathway inhibitor 3 (DKK3) in a dose-dependent manner. The immunohistochemistry confirmed the downregulation of DKK3 in tumor tissues. Moreover, DKK3 was correlated with FIGO stage and lymph node metastasis. Functionally, DKK3 overexpression significantly suppressed cell viability, colony formation and invasion, but promoted apoptosis in CaSki and HeLa cells. Overexpression of DKK3 upregulated the protein levels of cleaved caspase-3 and E-cadherin, but downregulated the protein levels of Bcl-2, N-cadherin and Vimentin. Furthermore, DKK3 knockdown reversed the suppressive effects of ApoG2 on CaSki cell proliferation, invasion and EMT markers, while DKK3 overexpression enhanced these effects. In addition, ApoG2 treatment inhibited CC xenograft tumor growth and upregulated the protein levels of DKK3, cleaved caspase-3 and E-cadherin. In conclusions, these findings suggested that ApoG2 could effectively inhibit the growth and invasion of CC cells at least partly by activating DKK3.

## Introduction

Cervical cancer (CC) is one of the most frequent and fatal gynecological malignancies overall the world, which is estimated to reach 460,000 annual deaths by 2040 (1, 2). The current treatment strategies for CC mainly focus on surgical resection, radiotherapy and chemotherapy, but they don’t always obtain satisfactory outcomes (3–5). For those patients unsuitable for surgery, some effective chemical drugs, including cisplatin, paclitaxel, 5-fluorouracila and doxorubicin have been applied for neoadjuvant chemotherapy (6). Unfortunately, drug resistance and toxic side effects remain a major cause of tumor recurrence (7). Thus, there is an urgent need to understand the molecular mechanisms of CC cells and develop effective non-toxic drugs for the prevention and treatment of CC.

Naturally occurring compounds have caused great attentions for the prevention of various cancers (8, 9), including CC (10). Gossypol, a yellow phenolic aldehyde, is identified as a natural polyphenol isolated from the seed, stem and roots of the cotton plant (*Gossypium*) that could provide the cotton plate with resistance to pests (11, 12). Gossypol was initially found to have anti-tumor effects against colon carcinoma and melanoma in 1984 (13). Subsequently, the antiproliferative activity of gossypol has been reported in prostate cancer (14), diffuse large cell lymphoma (15), and head and neck squamous cell carcinoma (16). However, the main adverse reactions of gossypol tablets included hypokalemia, muscle weakness, loss of appetite, nausea, vomiting and other gastrointestinal reactions, as well as palpitations and mild changes in liver function, which impede the suppressive efficacy of gossypol mainly due to two aldehyde groups in the molecule’s structure of gossypol (17). In recent years, Apogossypolone (ApoG2), a novel derivative of gossypol lacking of two aldehyde groups, was synthesized and identified as a small-molecule inhibitor of anti-apoptotic Bcl-2 proteins with much higher antitumor activity than gossypol (18). For example, He et al. (19) showed that ApoG2 effectively suppressed tumor growth of nasopharyngeal carcinoma (NPC) xenografts in nude mice and enhanced the antitumor effect of cisplatin on NPC cells *in vitro* and *in vivo*. ApoG2, could inhibit the antiapoptotic function of Bcl-2, Mcl-1, and Bcl-XL and induce apoptosis in pancreatic cancer cells (20). In addition, ApoG2 could inhibit tumor cell invasion and migration *via* a converse epithelial-to-mesenchymal transition (EMT) process in pheochromocytoma (21). Despite the antitumor activities of ApoG2 have been widely reported, little is known about the effects of ApoG2 in human CC.

Dickkopf Wnt signaling pathway inhibitor 3 (DKK3), localized on 11p15, is a potential tumor suppressor gene for the often deleted-locus in cancerous cells (22). Emerging evidence indicates DKK3 is downregulated in various cancer tissues, including renal clear cell carcinoma (23), non-small cell lung cancer (24) and uterine cervical squamous cell carcinoma (25). Functionally, overexpression of DKK3 can decrease cell invasion, proliferation, and colony forming ability in gallbladder cancer cells (26), colorectal cancer (27), and lung adenocarcinoma (28). What’s more, DKK3 belongs to a family of Wnt antagonists, and its downregulation and methylation have been reported in multiple malignancies (29–31). Interestingly, a previous report by Lee et al. (32) pointed that DKK3 was downregulated in CC and functioned as a negative regulator of beta-catenin signaling pathway. Furthermore, Lee et al. demonstrated that the reintroduction of DKK3 into HeLa CC cells resulted in reduced colony formation and retarded cell growth (32). In addition, DKK3 can inhibit Rac1-c-Jun N-terminal kinase (JNK) signaling and critical signaling pathways involved in CC cell proliferation and survival (33, 34). Nevertheless, whether ApoG2 regulated CC cell functions was associated with DKK3 expression has not been uncovered yet.

Hence, this study aimed to investigate the effects and mechanisms of ApoG2 on the CC *in vitro* and *in vivo*. We first analyzed the effects of ApoG2 (2.5, 5, 10 and 20 µmol/L) on cell viability, colony formation, apoptosis, invasion ability and EMT markers in CC cells. Moreover, we further explored whether ApoG2 induced these effects were correlated with DKK3 regulation *in vitro*. Furthermore, the antitumor activity of ApoG2 was also evaluated in CC tumor growth in nude mice.

## Materials and Methods

### Cell Culture and Treatment

Two CC cell lines, including CaSki and HeLa were obtained from American Type Culture Collection (ATCC, Rockville, MD, USA) and cultured in DMEM containing 10% FBS (Thermo Fisher Scientific, Waltham, MA, USA), streptomycin (100 μg/mL) and penicillin (100 IU/mL) in an incubator with 5% CO_2_ at 37°C. CaSki and HeLa were in cultures were respectively incubated with 0 (0.1% DMSO), 2.5, 5, 10 and 20 µmol/L ApoG2 (ApexBio Technology, Boston, MA) with culture medium for indicated times.

### Cell Transfection

The full-length cDNA encoding human DKK3 was amplified with primers (forward: 5′-ATCCGGGTTCGGTTGCTC-3′; reverse: 5′-GCAGCTTCTTCTGCCTCCAT-3′) and obtained PCR products were ligated into the pcDNA3.1 vector (Invitrogen, Carlsbad, CA, USA) to create the plasmid pcDNA3.1-DKK3. Meanwhile, small interring RNA targeting DKK3 (si-DKK3: 5′- GGAGGACACGCAGCACAAATT-3′) and negative control (si-NC: 5′-GGCCGAAGTCGAAGACCATAA-3′) were synthesized by GenePharma (Shanghai, China). After reached 80% confluence, CaSki and HeLa cells were transfected with pcDNA3.1-DKK3 or pcDNA3.1 vector for 48 h. In the rescue experiments, CaSki cells were transfected with si-DKK3 or pcDNA3.1-DKK3 for 48 h, followed by 20 µmol/L of ApoG2 treatment. Transfection of all plasmids and oligonucleotides were performed by Lipofectamine 3000 reagent (Invitrogen, USA).

### CCK-8 Assay

Cell viability was determined by performing Cell Counting kit-8 (CCK-8) assay. In brief, CaSki and HeLa cells from different groups at a density of 5,000 cells per well were seeded in 96-well plates and cultured overnight. At 24, 48, and 72 h, 10 μL CCK-8 solution (Dojindo Molecular Technologies, Inc., Kumamoto, Japan) was added into cells in each well. After incubation for another 2 h at 37°C, the optical density (OD) values of each well were measured at 450 nm using a microplate reader. All cell viability assays were replicated in triplicate.

### Colony Formation Assay

CaSki and HeLa cells from different groups were trypsinized and inoculated into six-well plates at a density of 300 cells per well in 2 mL media for colony formation assay. After cultured for two weeks, cells were fixed with 4% paraformaldehyde at 37°C with 5% CO_2_ and then stained with 0.1% crystal violet for 15 min at room temperature. The colonies containing a minimum number of 50 cells per colony were photographed and counted under a microscope.

### Detection of Apoptosis by Flow Cytometry

The apoptotic rate of CC cells was detected using flow cytometry with Annexin V-FITC/PI double staining (BD Biosciences, San Jose, CA, USA). Briefly, CaSki and HeLa cells from different groups were trypsinized, washed twice in cold PBS and resuspended in a binding buffer. Subsequently, cells were stained with 5 μL FITC-labeled Annexin V (1 μg/mL) and 5 μL propidium iodide (PI; 50 µg/mL) for 20 min. The apoptotic rate was determined by FACScan flow cytometry using the Cell Quest program (BD Biosciences).

### Transwell Invasion Assay

Matrigel invasion assay was performed in CC cells using transwell chambers (8 μm pore size; Millipore Corporation, Billerica, MA, USA). Before inoculation of CC cells, 60 μL FBS-free media diluted Matrigel (BD Biosciences) was covered in polycarbonate membrane of the upper chamber and placed in 24-well plates, which was incubated at 4°C for 10 min and further kept at 37°C for 1 h until gel was formed. Next, 200 μL CaSki and HeLa cell suspension in FBS-free media containing 5 × 10^4^ cells were seeded into the upper chamber. At the same time, the lower chamber was added with 500 μL media containing 15% FBS as a chemoattractant. After 24 h incubation with 5% CO_2_ at 37°C, invasive cells on the lower chamber were fixed with 4% paraformaldehyde for 30 min, washed thrice with tri-distilled water and stained with 0.1% crystal violet for 30 min. Subsequently, invasive cells were photographed using microscope (×100) and counted using Image‐pro Plus 6.0 (Media Cybernetics, Rockville, MD, USA).

### Clinical Samples

Total 51 cases of tumor tissues and matched adjacent non-cancerous tissues with clinical features were collected from CC patients at the department of Obstetrics and Gynecology of Qilu Hospital of Shandong University (Shandong, China) between January 2015 and December 2020. All patients did not receive any preoperative anti-tumor therapies, including radiotherapy, chemotherapy or immunotherapy and written informed consent was signed by each patient before tissue collection. This study was performed in accordance with Helsinki Declaration and approved by the Medical Ethics Committee of Qilu Hospital of Shandong University (Approval No. KYLL-2017-560; 2017.1.15; Shandong, China).

### Immunohistochemistry Assay

For immunohistochemical staining, tissue samples were routinely dehydrated and embedded in paraffin. Paraffin-embedded tissues were cut into 4-μm thickness sections and dewaxed with xylene for 5 min which was repeated three times. Following rehydration in graded alcohol, the sections were treated with heat-activated antigen retrieval for 15 min, followed by blocking of endogenous peroxidase in 3% hydrogen peroxide and methanol for 15 min at room temperature. Afterwards, the sections were incubated with primary antibody against DKK3 (1: 500, Abcam, Cambridge, MA, USA) at 4°C overnight, rinsed thrice with PBS for 5 min, and then incubated with streptavidin-horseradish peroxidase (HRP)-conjugated secondary antibody at room temperature for 1 h. Finally, the sections were visualized with 3,3-diaminobenzadine for 10 min, and counterstained with hematoxylin. Immunoreactivity was independently assessed by two board-certified pathologists in a blinded manner as previously described (35). In brief, the immunoreactivity score was calculated by multiplying the staining positivity on a scale of 0-3 (0, no staining; 1, weak; 2, moderate; 3, strong staining) and staining proportion (1, <10%; 2, 10%–24%; 3, 25%–49%; and 4, ≥50%). Immunoreactivity was then classified as weak staining (-+), moderate staining (+) and strong staining (++) with the score scaling 0-2, 2-4 and over 4, respectively. Moreover, all CC tissues were categorized into high DKK3 (score ≥ 4) and low DKK3 (score < 4) groups.

### Xenograft Tumor Assay

Five-week-old female BALB/c nude mice weighing 18-22 g were purchased from Beijing Vital River Laboratory Animal Technology Co., Ltd. (Beijing, China) and housed in pathogen-free and temperature-controlled conditions under a 12 h light/dark illumination cycle. The human CaSki cells were trypsinized, centrifuged for 4 min at 1,000 rpm, and adjusted to 3 × 10^7^ cell/mL with serum-free culture medium. A total of 500 μL cell suspension was inoculated subcutaneously into the back region of nude mice (3 × 10^6^ cells/mouse). When the tumor size reached ~4 mm in diameter, all mice were randomly divided into control (9% DMSO) and ApoG2-treatment (ApoG2 was suspended in 9% DMSO) groups with five mice each group. All pharmacologic agents were administered at a dose of 100 mg/kg by intraperitoneal injection every other day for 24 days. The antitumor activity of ApoG2 was assessed by the calculation of tumor volume based on the formula: tumor volume (mm^3^) = (L × W^2^)/2, where L and W represent the tumor length and width, respectively. At 24 days post-injection, mice were euthanized and tumors were dissected and weighed. All tissue samples were harvested for western blot analysis. All animal procedures were performed in accordance with institutional protocols approved by the Institutional Animal Care and Use Committee of Key Laboratory of Gynecologic Oncology of Shandong Province (Approval No. KGO-2018-241; 2018, 3.15; Shandong, China).

### Western Blot Analysis

Extraction of protein sample was performed with radio immunoprecipitation assay (RIPA) lysis buffer (Beyotime, Jiangsu, China). After analyzing protein concentration by a BCA protein assay kit (Beyotime, China), equal amount of protein (30 μg) was separated by 12% sodium dodecyl sulfate–polyacrylamide gel electrophoresis (SDS-PAGE) and then transferred onto polyvinylidene difluoride (PVDF; Millipore, USA) membranes. The PVDF membranes were blocked with phosphate buffer saline with 0.1% Tween-20 (PBST) for 2 h and then incubated with primary antibodies against DKK3 (ab126080), Bcl-2 (ab59348), Cleaved caspase-3 (ab2302), E-cadherin (ab238099), N-cadherin (ab76059), Vimentin (ab137321) and GAPDH (ab37168) (all from Abcam, Cambridge, MA, USA) overnight at 4°C. Following rinsed with PBST thrice, the membranes were incubated with a horseradish peroxidase-conjugated anti-IgG (SC-2054, Santa Cruz) as the secondary antibody, followed by visualization of protein bands by ECL chemiluminescent reagent (Pierce Biotechnology, Rockford, USA).

### Statistical Analysis

All statistical analysis was performed using GraphPad Prism 6.0 (GraphPad, CA, USA). Data obtained from three independent experiments were expressed as mean ± standard deviation (SD). Comparison between two groups was analyzed using the student’s t-test and comparison among multiple groups was analyzed using one-way analysis of variance, followed by Dunnett’s test. The Chi-squared test was used to evaluate the correlation of DKK3 expression and clinicopathological parameters. The *p*-value of less than 0.05 was considered to be statistically significant.

## Results

### ApoG2 Inhibited Cell Proliferation, Invasion and EMT Process in CC Cells, Along With Upregulation of DKK3 in a Dose-Dependent Manner

**Figure 1:**
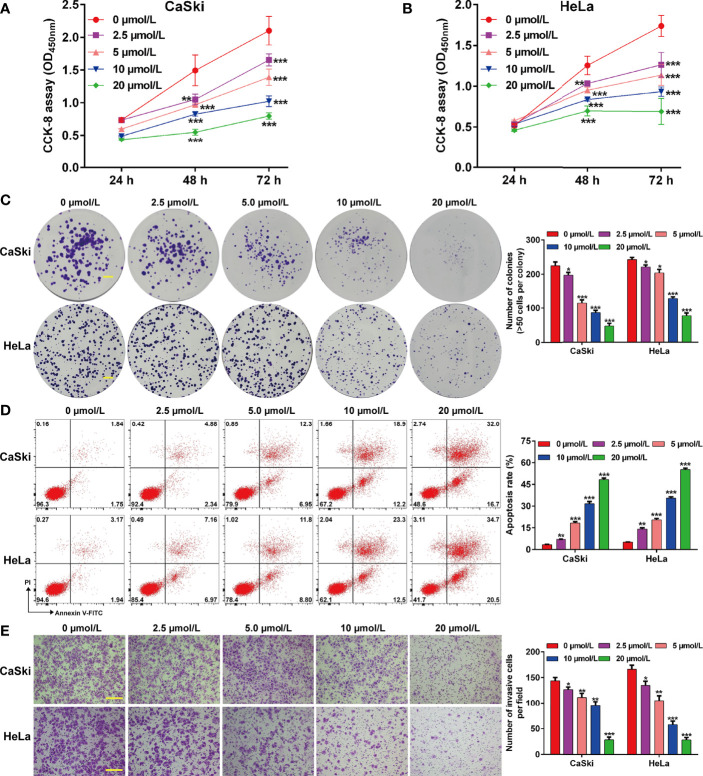
Effects of ApoG2 treatment on CC cell proliferation, apoptosis and invasion. CaSki and HeLa cells were treated with 0 (0.1% DMSO), 2.5, 5, 10 and 20 µmol/L ApoG2 for 24 h, respectively. **(A, B)** CCK-8 assay was used to analyze cell viability in treated CaSki and HeLa cells. **(C)** Representative photographs and quantitative analyses of plate colony formation of treated CaSki and HeLa cells. Scale bar, 50 μm; **(D)** The apoptotic rate of treated CaSki and HeLa cells was measured by flow cytometry. **(E)** Treated CaSki and HeLa cells that invaded through the microporous membrane were shown in photographs (left panel) and the numbers of invasive cells were shown in a histogram (right panel). Scale bar, 100 μm; Data are expressed as means ± SD. *p < 0.05, **p < 0.01, ***p < 0.001, compared with 0 µmol/L ApoG2; ApoG2, apogossypolone; CC, cervical cancer.

**Figure 2:**
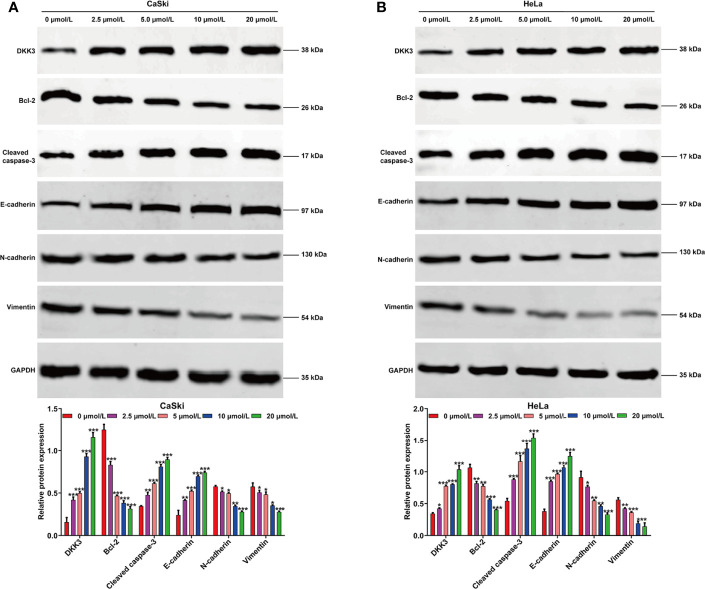
Effects of ApoG2 treatment on DKK3 expression and protein markers of apoptosis and EMT process in CC cells. CaSki and HeLa cells were treated with 0 (0.1% DMSO), 2.5, 5, 10 and 20 µmol/L ApoG2 for 24 h, respectively. Western blot analysis was performed to detect the protein levels of DKK3, Bcl-2, cleaved caspase-3, E-cadherin, N-cadherin and Vimentin in treated CaSki **(A)** and HeLa **(B)** cells. Data are expressed as means ± SD. *p < 0.05, **p < 0.01, ***p < 0.001, compared with 0 µmol/L ApoG2; ApoG2, apogossypolone; CC, cervical cancer.

To investigate the effects of ApoG2 on CC cells, two CC cell lines (CaSki and HeLa) were treated with different dosages of ApoG2 (0, 2.5, 5, 10 and 20 µmol/L). The results from CCK-8 assay showed that ApoG2 significantly decreased the viability of human CaSki (**Figure 1A**) and HeLa (**Figure 1B**) cells in a dose-dependent manner. Similarly, the number of colonies was remarkedly reduced with the increased concentration of ApoG2 treatment in CaSki and HeLa cells (**Figure 1C**). Consistent with the cell proliferation trend, flow cytometry analysis demonstrated that ApoG2 significantly promoted the apoptotic rate of CaSki and HeLa cells in a dose-dependent manner (**Figure 1D**). In addition, transwell invasion assay indicated that the number of invasive cells was markedly decreased from control group (0 µmol/L ApoG2) to increased concentration of ApoG2 treatment in CaSki and HeLa cells (**Figure 1E**). Furthermore, the results from western blot analysis further confirmed that ApoG2 upregulated the expression of DKK3 in a dose-dependent manner, implying DKK3 might play an important role in CC cell behaviors. In line with the effects of ApoG2 on cell apoptosis and invasion, ApoG2 obviously upregulated the protein levels of caspase-3 and E-cadherin, while downregulated the protein levels of Bcl-2, N-cadherin and Vimentin in a dose-dependent manner in both CaSki (**Figure 2A**) and HeLa (**Figure 2B**) cells.

### The Expression of DKK3 Was Downregulated in CC Tissues

**Figure 3:**
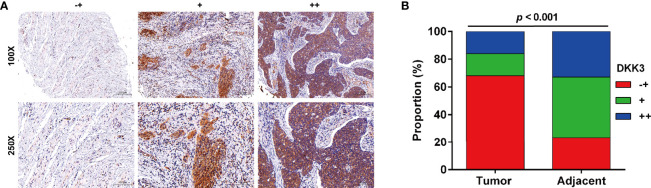
Expression of DKK3 in CC tissues. **(A)** Representative immunostaining of DKK expression in adjacent and CC samples (-+, weak staining, + moderate staining, ++ strong staining). **(B)** Summary of DKK3 expression in adjacent and CC samples. Blue represents ++ strong staining; Red represents -+, weak staining; Green represents + moderate staining; Magnification, ×250; scale bar, 100 μm; Magnification, ×100; scale bar, 200 μm.

Next, we analyzed the protein expression of DKK3 in CC in collected 51 paired tumor and adjacent tissues by performing immunohistochemical staining. Representative photomicrographs of different DKK3 protein staining degrees were depicted in **Figure 3A**. Statistical analysis further indicated increased weaker staining of DKK3 protein in paraffin-embedded tumor tissues, in comparison with that in adjacent tissues (**Figure 3B**). By contrast, we observed significantly decreased moderate and strong staining of DKK3 protein in tumor tissues compared with adjacent tissues. According to immunoreactivity score, all the CC patients were divided into high (n = 19) and low (n = 32) DKK3 expression groups. The results from Chi-squared test (**Table 1**) showed that the expression level of DKK3 was significantly associated with FIGO stage (*p* = 0.011) and lymph node metastasis (*p* = 0.014).

**Table 1:** Association between DKK3 expression and clinicopathological characteristics of cervical cancer patients.

Characteristics	Cases (n = 51)	Expression of DKK3		*p*-value
		Low (n = 32)	High (n = 19)	(chi-square test)
**Age**				0.581
< 45 years	18	11	8	
≥ 45 years	33	21	11	
**Tumor size**				0.306
< 4 cm	34	23	11	
≥ 4 cm	17	9	8	
**FIGO stage**				0.011*
I	38	20	18	
II-III	13	12	1	
**Histologic type**				0.250
Adenocarcinoma	19	10	9	
Squamous cell carcinoma	32	22	10	
**Lymph node metastasis**				0.014*
Negative	29	14	15	
Positive	22	18	4	
**Lymphatic vascular infiltration**				0.611
Negative	37	24	13	
Positive	14	8	6	
**Differentiation**				0.232
Well/moderate	27	19	8	
Poor	24	13	11	

FIGO, international federation of gynecology and obstetrics; DKK3, Dickkopf homolog 3.

*p < 0.05.

### Overexpression of DKK3 Suppressed Cell Proliferation, Invasion and EMT Process in CC Cells

**Figure 4:**
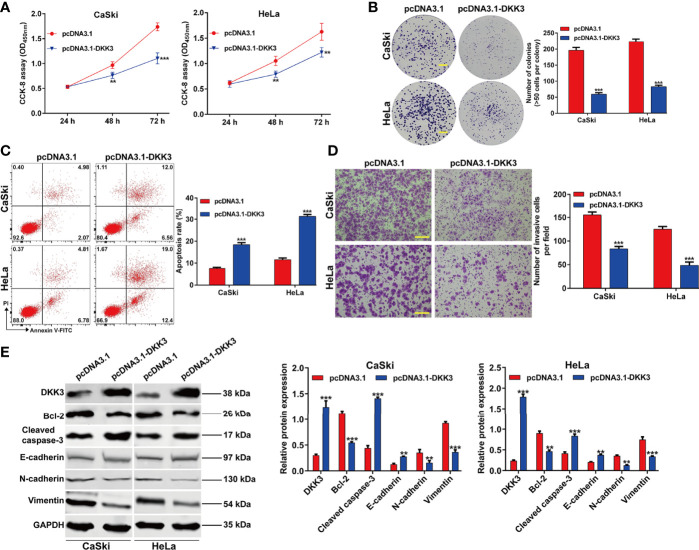
Effects of DKK3 overexpression on cell proliferation, apoptosis and EMT process in CC cells. CaSki and HeLa cells were transfected with pcDNA3.1-DKK3 or pcDNA3.1 for 48 h. **(A)** CCK-8 assay was used to analyze cell viability in transfected CaSki and HeLa cells. **(B)** Representative photographs and quantitative analyses of plate colony formation of transfected CaSki and HeLa cells. Scale bar, 50 μm; **(C)** The apoptotic rate of transfected CaSki and HeLa cells was measured by flow cytometry. **(D)** Cell invasion was assessed by transwell assay in transfected CaSki and HeLa cells and the numbers of invasive cells were shown in a histogram. Scale bar, 100 μm; **(E)** Western blot analysis was performed to detect the protein levels of DKK3, Bcl-2, cleaved caspase-3, E-cadherin, N-cadherin and Vimentin in transfected CaSki and HeLa cells. Data are expressed as means ± SD. *p < 0.05, **p < 0.01, ***p < 0.001, compared with pcDNA3.1.

To confirm the functional role of DKK3 in CC *in vitro*, we performed gain-of-function assays in CaSki and HeLa cells. Firstly, transfection of CaSki and HeLa cells with pcDNA3.1-DKK3 resulted in a significant decrease in cell viability, compared with pcDNA3.1 transfection (**Figure 4A**). The results from colony formation assay consistently indicated that the number of colonies was obviously reduced in pcDNA3.1-DKK3 group, compared with pcDNA3.1 group in CaSki and HeLa cells (**Figure 4B**). Moreover, a significantly increased apoptotic rate was observed in CaSki and HeLa cells after DKK3 overexpression (**Figure 4C**). Transwell assay demonstrated that overexpression of DKK3 remarkedly suppressed cell invasive ability of CaSki and HeLa cells (**Figure 4D**). After confirmed the upregulation of DKK3, we further manifested that anti-apoptotic Bcl-2 was downregulated and pro-apoptotic caspase-3 was upregulated in CaSki and HeLa cells following pcDNA3.1-DKK3 transfection (**Figure 4E**). The suppressive effects of DKK3 overexpression on EMT process was also confirmed, as reflected by increased E-cadherin expression and decreased protein levels of N-cadherin/Vimentin in CaSki and HeLa cells (**Figure 4E**).

### ApoG2 Suppressed CC Cell Proliferation, Invasion and EMT Process by Upregulating DKK3

**Figure 5:**
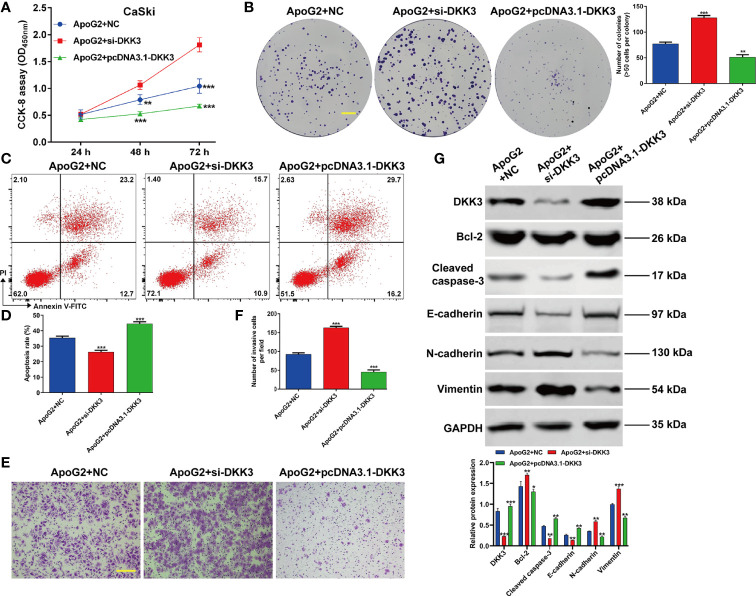
ApoG2 suppressed CC cell proliferation, invasion and EMT process by upregulating DKK3. CaSki cells were transfected with pcDNA3.1-DKK3, si-DKK3 or NC, followed by treatment with 20 µmol/L ApoG2. **(A)** CCK-8 assay was used to analyze cell viability in transfected CaSki cells. **(B)** Representative photographs and quantitative analyses of plate colony formation of transfected CaSki cells. Scale bar, 50 μm; **(C, D)** The apoptotic rate of transfected CaSki cells was measured by flow cytometry. **(E, F)** Cell invasion was assessed by transwell assay in transfected CaSki cells and the numbers of invasive cells were shown in a histogram. Scale bar, 100 μm; **(G)** Western blot analysis was performed to detect the protein levels of DKK3, Bcl-2, cleaved caspase-3, E-cadherin, N-cadherin and Vimentin in transfected CaSki cells. Data are expressed as means ± SD. *p < 0.05, **p < 0.01, ***p < 0.001, compared with ApoG2 + NC.

Subsequently, we further explored that whether the suppressive effects of ApoG2 in CC cells were correlated with upregulation of DKK3 expression. At first, CaSki cells were transfected with pcDNA3.1-DKK3, si-DKK3 or NC, followed by treatment with 20 µmol/L ApoG2. Through CCK-8 assay (**Figure 5A**) and colony formation assay (**Figure 5B**), we observed that the inhibitory activity of ApoG2 on cell viability and colony formation was reversed by DKK3 knockdown, but significantly enhanced by DKK3 overexpression in CaSki cells. Flow cytometry demonstrated that elevated apoptotic rate of CaSki cells induced by ApoG2 treatment was attenuated after DKK3 knockdown, while further increased after DKK3 overexpression (**Figures 5C, D**). Additionally, knockdown of DKK3 promoted the cell invasion ability in ApoG2-treated CaSki cells, which was reversed after DKK3 overexpression (**Figures 5E, F**). At molecular level, the protein levels of DKK3, Bcl-2, caspase-2, E-cadherin, N-cadherin and Vimentin in ApoG2-treated CaSki cells were evidently abolished after DKK3 knockdown, but promoted by DKK3 overexpression (**Figure 5G**). Taken together, these data suggested that ApoG2 exhibited the anti-tumor activity *in vitro* may through upregulating DKK3 expression.

### ApoG2 Suppressed Tumor Growth in CaSki Xenograft in the BALB/c Nude Mice

**Figure 6:**
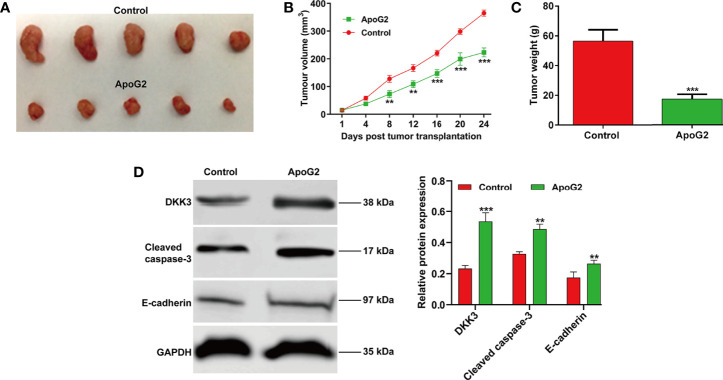
ApoG2 inhibited tumor growth *in vivo*. **(A)** Image showing human CaSki tumor xenografts removed from the BALB/c nude mice (n = 5 each group). **(B)** Tumor volume growth curves and **(C)** tumor weights for ApoG2 treatment and control groups. **(D)** The protein levels of DKK3, cleaved caspase-3 and E-cadherin in ApoG2-treated CaSki xenografts and control groups. Data are expressed as means ± SD. **p < 0.01, ***p < 0.001, compared with control.

Having investigating the effect of ApoG2 on CC cell proliferation *in vitro*, we next studied the effect of ApoG2 on tumorigenesis in a nude mouse model. The *in vivo* study of subcutaneously inoculated CaSki cells in BALB/c-nu mice showed that ApoG2 treatment could slow down mice tumor growth compared with control group (**Figure 6A**). After quantification analysis, we found that the tumor volume (**Figure 6B**) and weight (**Figure 6C**) in ApoG2 group were significantly decreased, compared with those in control group. Subsequently, western blot analysis was performed to examine related protein markers. As shown in **Figure 6D**, the upregulation of DKK3, cleaved caspase-3 and E-cadherin was confirmed in CaSki xenograft-bearing nude mice treated with ApoG2, when compared with those treated with controls. Based on these results, the ApoG2 treatment was demonstrated to suppress tumor growth in CC xenografts.

## Discussion

In the present study, we chose ApoG2, the most potent derivative of gossypol with high affinity against anti-apoptotic Bcl-2 family members (pan-Bcl-2 inhibitor), including Bcl-2, Bcl-XL and Mcl-1 in tumor-targeted treatment (36). Data presented in **Figure 1** revealed that ApoG2 could effectively inhibit cell viability, colony formation and invasion, but promote apoptotic rate of CaSki and HeLa cells in a dose-dependent manner. Moreover, ApoG2 treatment caused a significant decrease in Bcl-2 expression and a remarkable increase in cleaved caspase-3 expression in a dose-dependent manner. As our best knowledge, ApoG2 is more stable and less toxicity than its parental compound gossypol, which was previously confirmed by Kitada et al. (37) who demonstrated that treatment with gossypol caused a great deal of hepatotoxicity and gastrointestinal toxicity to the animals compared to treatment with vehicle control or ApoG2. A recent report by Zhang et al. (20) indicated that ApoG2 was significantly more tolerable than gossypol in rats and mice. Similar to our data, ApoG2 could effectively inhibit proliferation of NPC cells that expressed anti-apoptotic Bcl-2 family proteins at a high level (19). The report by Zheng et al. (38) further supported the suppressive role of ApoG2 on the growth of human NPC cells by inducing apoptosis and autophagy. ApoG2 could induce apoptosis through up-regulation of Bax and down-regulation of Bcl-2, increasing reactive oxygen species (ROS) levels, inducing cytochrome C release and cleaving caspase proteins (39). In addition, ApoG2 inhibits the growth and proliferation of gastric cancer cells by down-regulating of Bcl-2 protein expression, up-regulating of Bax and activating of caspase-3 (40). Based on these evidences, we thus concluded that ApoG2 could trigger mitochondrial-dependent CC cell apoptosis.

In addition to apoptosis indued by ApoG2, our data showed that ApoG2 could effectively suppressed the invasion ability of CaSki and HeLa cells, along with impaired EMT process (increased E-cadherin and decreased N-cadherin/vimentin) in a dose-dependent manner. During embryonic development, epithelial-to-mesenchymal transition (EMT) is a normal process but is abnormally reactivated during tumor progression, causing tumor acquires motility properties (41). Tumor migration and invasion is positively correlated with the development of EMT process, as reflected by the loss of epithelial markers (E-cadherin and β-catenin) on the membrane and the acquisition of mesenchymal markers (vimentin, Twist and Snail) (42). In line with our data, Lin et al. (21) demonstrated that ApoG2 inhibited cell mobilities *via* promotion of E-cadherin and β-catenin translocation from cytoplasm to membrane dependent on the downregulation of the PI3K/AKT pathway in malignant pheochromocytoma. These findings suggested that ApoG2 may have a therapeutic potential as a new class of anti-CC agents.

The data from western blot analysis additionally pointed that DKK3 expression was downregulated in CaSki and HeLa cells after ApoG2 treatment in a dose-dependent manner. Furthermore, we further confirmed the decreased DKK3 expression in CC tissues using related clinical samples, which was significantly associated with FIGO stage (*p* = 0.011) and lymph node metastasis (*p* = 0.014). In fact, DKK3 acts as a tumor suppressor by inhibiting tumor cell proliferation, migration and invasion, and promoting apoptosis in gallbladder cancer cells (26), colorectal cancer (27), and lung adenocarcinoma (28). Here, we performed gain-of-function assay to show that DKK3 overexpression significantly inhibited cell viability, colony formation and invasion, but promoted apoptotic rate in CaSki and HeLa cells. Consistently, the role of DKK3 on CC has been previously reported through the following articles: Ryu et al. (25) pointed that decreased DKK3 expression was associated with advanced International Federation of Gynecology and Obstetrics clinical stages, which was predictive of lower disease-free survival in patients with cervical squamous cell carcinoma. Lee et al. (32) showed that DKK3 is a negative regulator of beta-catenin and its downregulation contributes to an activation of the beta-catenin signaling pathway in CC. Luo et al. (43) additionally manifested that DKK3 as the direct downstream regulator was involved in the promotive effects of miR-93a in CC cell viability and invasion. Most importantly, our data further confirmed that ApoG2 suppressed CC cell proliferation, invasion and EMT process, as well as tumor growth by upregulating DKK3. Combined with the similar suppressive effects of ApoG2 and DKK3 on CC, we thus not hard to conclude that DKK3 might be a downstream regulator participated in ApoG2 exerting its tumor suppressive effects in CC *in vitro* and *in vivo*. However, the exactly molecular mechanism underlying ApoG2 regulating DKK3 in CC still needed to be further explored.

Of course, there were some limitations in our study as follows: 1) Lacking of high sequencing analysis to figure out the promising mechanism after administration with ApoG2 and the target genes about the protective effects during the poor progression of CC; 2) Lacking of relevant technologies of Genome Editing and Luciferase to make the explanation about the detailed mechanism is modulating DKK3; 3) Lacking of rescue experiments on HeLa cells *in vitro*.

Collectively, the present study demonstrated that ApoG2 exerted significantly suppressive effects on CC cell proliferation, invasion and EMT process *in vitro* and *in vivo*. What’s more, we identified that DKK3 might be a downstream effector of ApoG2 exerting tumor suppressive effects in CC. Even though future studies need to elucidate DKK3 or partner and the associated signaling pathway, our data to some degree represents a viable drug candidate for the development of novel anti-cancer therapies.

## Data Availability Statement

The raw data supporting the conclusions of this article will be made available by the authors, without undue reservation.

## Ethics Statement

The studies involving human participants were reviewed and approved by Qilu Hospital of Shandong University (Approval No. KYLL-2017-560; 2017.1.15; Shandong, China). The patients/participants provided their written informed consent to participate in this study. The animal study was reviewed and approved by The Institutional Animal Care and Use Committee of Key Laboratory of Gynecologic Oncology of Shandong Province (Approval No. KGO-2018-241; 2018, 3.15; Shandong, China).

## Author Contributions

YZ conceived the present study. YL made substantial contributions to conception and design. YL and JQ designed, performed and interpreted the experimental data. LL and YS analyzed the data. YL and JZ drafted the initial manuscript. JZ and SH confirm the authenticity of all the raw data. All authors have read and approved the final manuscript.

## Funding

This work was supported by The Key Research Project of Shandong Province (2017CXGC1210) and Jinan City “20 New Universities” independent innovation group (2021GXRC027).

## Conflict of Interest

The authors declare that the research was conducted in the absence of any commercial or financial relationships that could be construed as a potential conflict of interest.

## Publisher’s Note

All claims expressed in this article are solely those of the authors and do not necessarily represent those of their affiliated organizations, or those of the publisher, the editors and the reviewers. Any product that may be evaluated in this article, or claim that may be made by its manufacturer, is not guaranteed or endorsed by the publisher.

## Abbreviations

CC, cervical cancer; ApoG2, Apogossypolone; DKK3, Dickkopf homolog 3; CCK-8, Cell Counting Kit-8; EMT, epithelial-to-mesenchymal transition; DMSO, dimethyl sulfoxide; ATCC, American Type Culture Collection; OD, optical density; RIPA, radio immunoprecipitation assay; SDS-PAGE, sodium dodecyl sulfate–polyacrylamide gel electrophoresis; PVDF, polyvinylidene difluoride; PBST, phosphate buffer saline with 0.1% Tween-20; SD, standard deviation.
