# Supplementary figures and images for: Apogossypolone Inhibits Cell Proliferation and Epithelial-Mesenchymal Transition in Cervical Cancer via Activating DKK3
Source: Front Oncol. 2022 Jul 18;12:948023. doi: 10.3389/fonc.2022.948023 (PMC9341244; doi:10.3389/fonc.2022.948023)

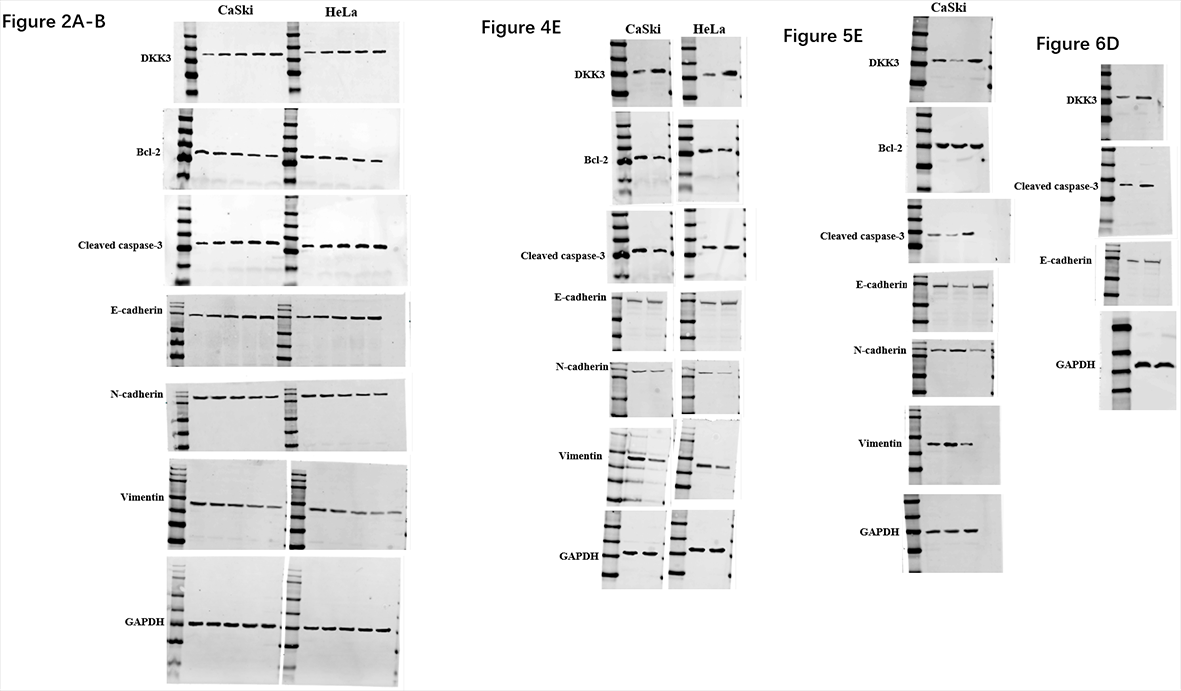

Supplement: Supplementary file 1 [file Image_1.tif]

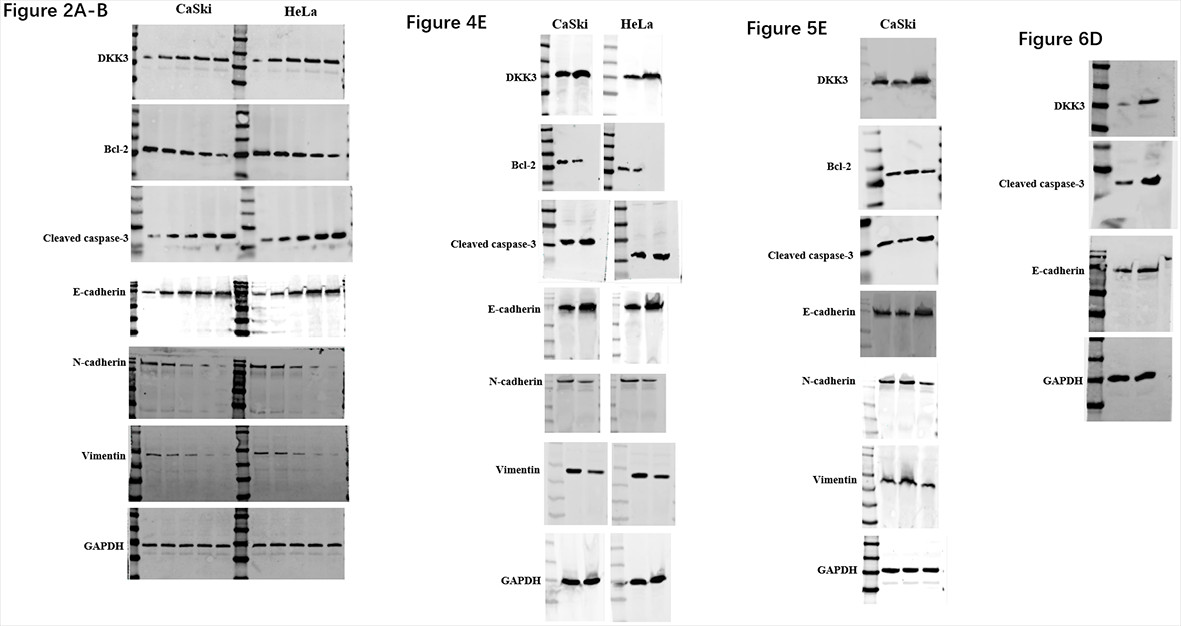

Supplement: Supplementary file 2 [file Image_2.tif]

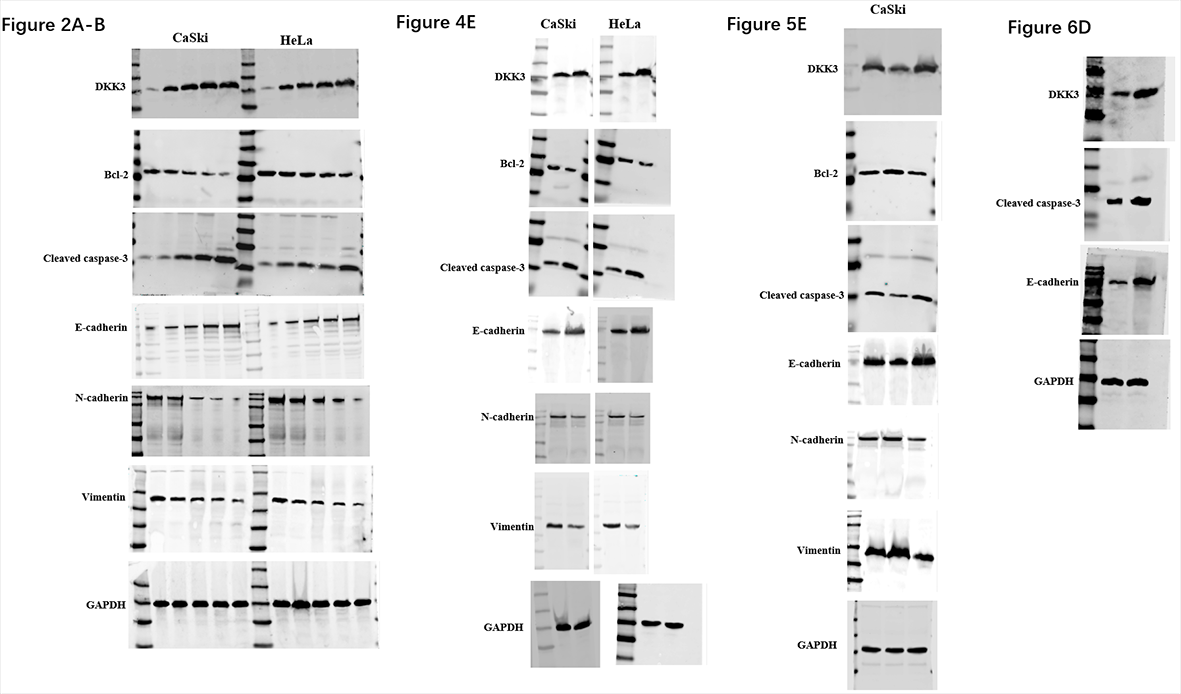

Supplement: Supplementary file 3 [file Image_3.tif]
